# Recurrence‐free survival dynamics following adjuvant chemotherapy for resected colorectal cancer: A systematic review of randomized controlled trials

**DOI:** 10.1002/cam4.6884

**Published:** 2024-01-08

**Authors:** Emma Vail, Ankur P. Choubey, H. Richard Alexander, David A. August, Abril Berry, Patrick M. Boland, Mariam F. Eskander, Miral S. Grandhi, Brittany Haliani, Haejin In, Timothy J. Kennedy, Russell C. Langan, Jason C. Maggi, Henry A. Pitt, Shridar Ganesan, Brett L. Ecker

**Affiliations:** ^1^ Division of Surgical Oncology Rutgers Cancer Institute of New Jersey, Rutgers Health New Brunswick New Jersey USA; ^2^ Rutgers Robert Wood Johnson University Medical School New Brunswick New Jersey USA; ^3^ Cooperman Barnabas Medical Center Livingston New Jersey USA; ^4^ Division of Medical Oncology Rutgers Cancer Institute of New Jersey, Rutgers Health New Brunswick New Jersey USA

**Keywords:** adenocarcinoma, adjuvant therapy, chemotherapy, colon cancer, rectal cancer, recurrence

## Abstract

**Background:**

Several cytotoxic chemotherapies have demonstrated efficacy in improving recurrence‐free survival (RFS) following resection of Stage II–IV colorectal cancer (CRC). However, the temporal dynamics of response to such adjuvant therapy have not been systematically quantified.

**Methods:**

The Cochrane Central Register of Trials, Medline (PubMed) and Web of Science were queried from database inception to February 23, 2023 for Phase III randomized controlled trials (RCTs) where there was a significant difference in RFS between adjuvant chemotherapy and surgery only arms. Summary data were extracted from published Kaplan–Meier curves using DigitizeIT. Absolute differences in RFS event rates were compared at matched intervals using multiple paired *t*‐tests.

**Results:**

The initial search yielded 1469 manuscripts. After screening, 18 RCTs were eligible (14 Stage II/III; 4 Stage IV), inclusive of 16,682 patients. In the absence of adjuvant chemotherapy, the greatest rate of recurrence was observed in the first year (mean RFS event rate; 0–0.5 years: 0.22 ± 0.21; 0.5–1 years: 0.20 ± 0.09). Adjuvant chemotherapy was associated with significant decreases in the RFS event rates for the intervals 0–0.5 years (0.09 ± 0.09 vs. 0.22 ± 0.21, *p* < 0.001) and 0.5–1 years (0.14 ± 0.11 vs. 0.20 ± 0.09, *p* = 0.001) after randomization, but not at later intervals (1–5 years). In Stage IV trials, RFS event rates significantly differed for the interval 0–0.5 years (*p* = 0.012), corresponding with adjuvant treatment durations of 6 months. In Stage II/III trials, which included therapies of 6–24 months duration, there were marked differences in the RFS event rates between surgery and chemotherapy arms for the intervals 0–0.5 years (*p* < 0.001) and 0.5–1 years (*p* < 0.001) with smaller differences in the RFS event rates for the intervals 1–2 years (*p* = 0.012) and 2–3 years (*p* = 0.010).

**Conclusions:**

In a systematic review of positive RCTs comparing adjuvant chemotherapy to surgery alone for Stage II–IV CRC, observed RFS improvements were driven by early divergences that occurred primarily during active cytotoxic chemotherapy. Late recurrence dynamics were not influenced by adjuvant therapy use. Such observations may have implications for the use of chemotherapy for micrometastatic clones detectable by cell‐free DNA‐based methodologies.

## Graphical abstract

In order to evaluate the temporal recurrence dynamics of colorectal cancer, we conducted a systematic review of Phase III randomized controlled trials centered on Stage II–IV disease, finding that adjuvant cytotoxic chemotherapy improved early—but not remote—recurrence event rates over surgery‐only controls. These results support the role of initially‐dormant micrometastatic populations in disease relapse, and therefore the ongoing evaluation of cell‐free DNA technologies to guide adjuvant treatment decisions and novel therapeutic strategies.

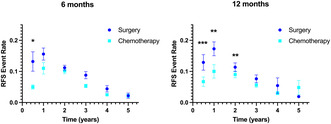

## 1 INTRODUCTION

Surgical resection is potentially curative for the management of colorectal cancer (CRC). However, the recurrence rate following surgery may be as high as 30% for patients with American Joint Commission on Cancer (AJCC) pathologic Stage II–III CRC and 80% for patients with resected AJCC Stage IV CRC.^1^, ^2^ Cancer recurrences are believed to arise from populations of micrometastatic clones present at the time of surgery. These manifest clinically within months of surgical resection or after more extended periods of disease remission—with disease relapse observed years or even decades after apparently curative resection.^3^ One of the predominant biological theories of cancer recurrence posits that relapse, even following years of remission, can occur when previously dormant, malignant cells stochastically reenter the cell cycle. Such cells may be present near the original site of resection, or disseminated to distant organs, and remain at subclinical levels throughout remission. This pause in cancer progression is thought to be a result of cells entering a dormant state, wherein growth is arrested yet fundamental survival pathways remain functional. The exact mechanisms of this dormancy are yet to be fully elucidated. It may involve interactions between quiescent cell and its microenvironment, with suppression of growth and altered cellular transcription/translation signals marked by stemness, autophagy, chemoresistance, and EMT.^4^, ^5^, ^6^, ^7^

Adjuvant chemotherapy is recommended for high‐risk AJCC Stage II and Stage III–IV CRC patients to reduce the risk of relapse. Typical regimens involve cytotoxic drugs that target the cell cycle, leading to irreversible growth arrest and cell death.^8^ Importantly, those residual micrometastatic populations that are in growth arrest (dormant cells that have exited the cell cycle) may prove resistant to cytotoxic chemotherapy. Several studies have indeed demonstrated that dormant tumor cells are refractory to traditional cytotoxic regimens.^6^, ^9^ Thus, adjuvant cytotoxic chemotherapy may prove efficacious in eliminating concurrently proliferating tumor cells but may not mitigate the risk of relapse mediated by the re‐initiation of growth in once‐quiescent subpopulations. Such a model predicts that adjuvant cytotoxic chemotherapy alters early recurrence rates by eradication of proliferating micrometastatic cells, but does not impact on late recurrence driven by the re‐emergence of dormant clones that persist in a chemorefractory state. To evaluate the validity of this hypothesis while testing the real‐world implications of dormancy‐induced resistance, we systemically reviewed the temporal dynamics of recurrence in response to adjuvant chemotherapy in published Phase III randomized controlled trials (RCTs).

## 2 MATERIALS AND METHODS

A systematic review was performed to identify all Phase III RCTs where there was a significant difference in recurrence‐free survival (RFS) between trial arms of curative‐intent surgery with subsequent adjuvant cytotoxic chemotherapy, and surgery alone with subsequent postoperative observation. Surgery was chosen as the control arm to allow for description of recurrence dynamics in the absence of all adjuvant therapy, thus describing the natural history of recurrence for resected CRC. All adjuvant treatment regimens were allowed, with pre‐planned analyses for type and duration of treatment.

RCTs that did not demonstrate a statistically significant improvement in RFS were excluded. While previous meta‐analyses have quantified the pooled hazard ratio for adjuvant therapy for RFS, this hypothesis‐generating review was instead uniquely focused on RFS dynamics among active regimens (i.e., once a regimen is demonstrated to be effective in improving RFS, at what time course during or after treatment does it lead to RFS improvements).

This systematic review is reported in accordance with the Preferred Reporting Items for Systematic Reviews and Meta‐Analyses (PRISMA) guidelines.^10^ A protocol for this systematic review is available from PROSPERO (CRD42023413791).

### 2.1 Search strategy and selection criteria

Two professional medical librarians (AB; BH) designed and executed the search strategy after input from the lead author (B.L.E.). The following databases were queried from database inception to February 23, 2023: Medline (PubMed), Web of Science, and the Cochrane Central Register of Trials. Standardized terms, keywords, and controlled vocabulary were combined to reflect the following concepts: colorectal adenocarcinoma, adjuvant chemotherapy, and randomized controlled trial. The search strategy is provided in the Data S1. Publications were screened for relevance by reading the title and abstract then subsequently the remainder of the manuscript if the former proved potentially informative. Bibliographies from RCTs of included studies were also searched to identify additional citations. Only English‐language publications were included. Two independent reviewers (E.V. and B.L.E.) screened all citations, and a third reviewer (S.G.) resolved discrepancies. After screening, two independent reviewers (E.V. and B.L.E) evaluated the full text of studies to identify RCTs for inclusion eligibility. Inclusion criteria were: (1) patients with resected Stage II–IV CRC; (2) control group of patients who received no adjuvant chemotherapy; (3) experimental group of patients who received adjuvant chemotherapy; (4) adequate statistics reported, including RFS and the corresponding Kaplan–Meier curves; and (5) a significant improvement in RFS with adjuvant chemotherapy, where statistical significance was defined individually by each trial.

Abstracts, duplicate articles, case reports, Phase I or II trials, unpublished manuscripts, non‐randomized studies, and trials utilizing perioperative chemotherapy were excluded. If multiple trials included overlapping cohorts, the study with the longest follow‐up or largest sample size was included. No assessment of bias or heterogeneity was performed.

### 2.2 Data analysis

RFS probabilities at pre‐specified times intervals from date of surgery were extracted from the published Kaplan–Meier curves using DigitizeIT (https://www.digitizeit.xyz/). The absolute differences in recurrence event rates (per trial arm) were calculated for half‐year intervals for the first year and 1‐year intervals between Years 1 and 5 (i.e., 0–0·5, 0·5–1, 1–2, 2–3, 3–4, and 4–5); this measurement (i.e., change in recurrence proportion over time) reflects the slope of the Kaplan–Meier curves at the prespecified time intervals. The absolute differences between treatment and control recurrence events at matched time intervals were compared using paired *t*‐tests. Planned analyses included all pooled trials, subsets grouped by AJCC stage (Stage II/III patients; Stage IV patients) and treatment duration (6 months; 12 months). *p*‐values less than or equal to 0.05 were considered statistically significant.

## 3 RESULTS

### 3.1 Trial characteristics

**FIGURE 1:**
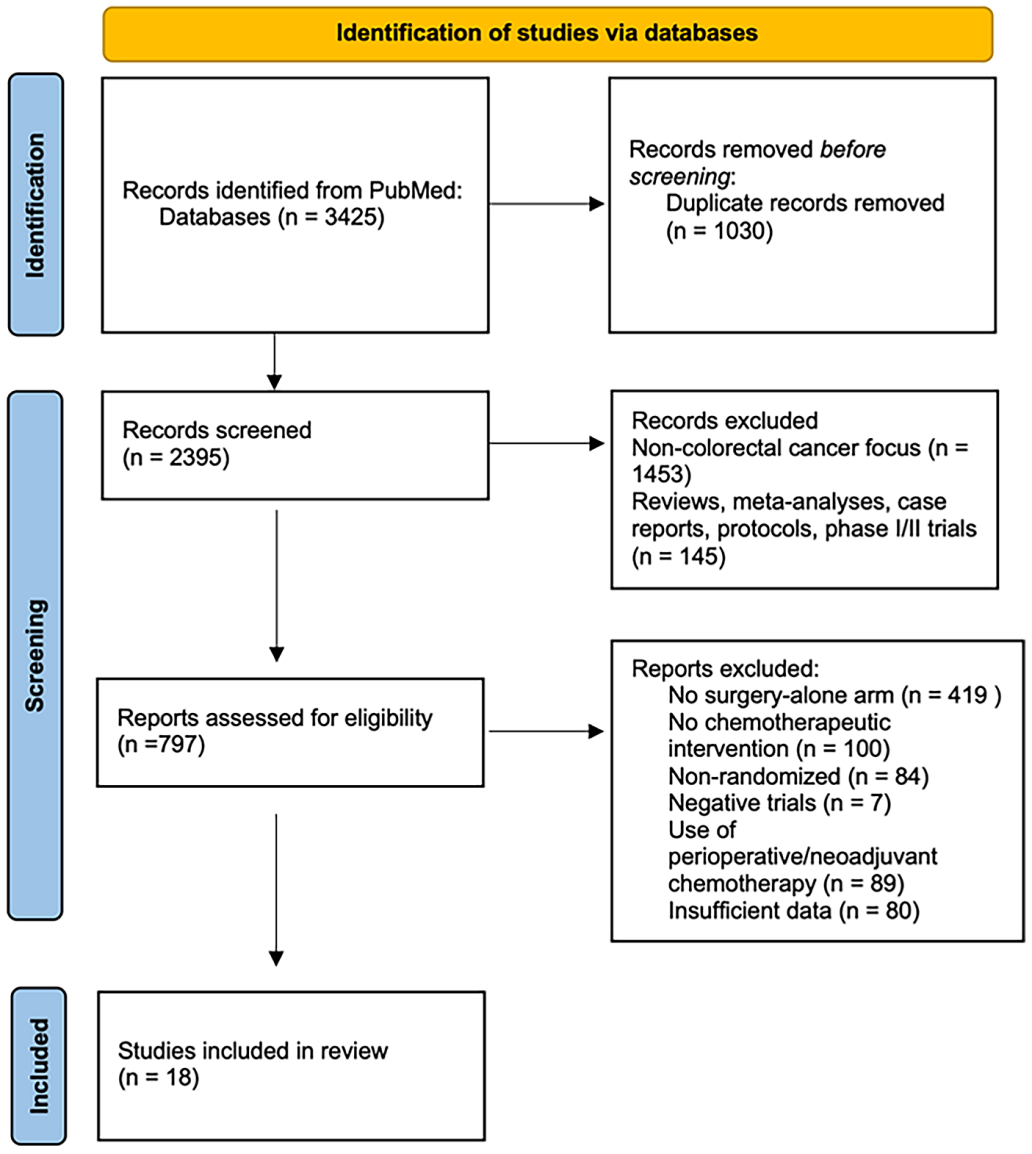
CONSORT of trials included in the systematic review.

The search strategy yielded 3425 citations. After screening 2395 citations, 797 articles met the inclusion criteria; among these, 18 unique RCTs with 16,682 patients were included in our systematic review.^11^, ^12^, ^13^, ^14^, ^15^, ^16^, ^17^, ^18^, ^19^, ^20^, ^21^, ^22^, ^23^, ^24^, ^38^, ^39^, ^40^, ^41^ This included four trials involving Stage IV CRC and 14 involving Stage II/III CRC. Figure 1 highlights the PRISMA flowchart for this study.

The median weighted follow‐up from randomization was 59.1 months (range 36–87 months). Therapeutic agents were varied, but generally consisted of fluorouracil, uracil‐tegafur (UFT), or carmofur (HCFU), often in combination with leucovorin, oxaliplatin, mitomycin, and/or levamisole. Chemotherapy treatment durations ranged from 6 months to 2 years. Table 1 summarizes the study characteristics of individual RCTs.

**TABLE 1:** Summary of included trials in meta‐analysis.

First author	Publication year	Treatment	*N*	*N* (surgery)	*N* (chemo)	HR (95% CI)
Stage II–III						
Hamaguchi^11^	2011	UFT; 52 weeks	274	135	139	0.66 (0.45–0.97)
Quasar Collective Group^12^	2007	5‐FU/LV; 24 weeks	3239	1617	1622	0.78 (0.67–0.91)
Watanabe^13^	2006	5‐FU/MMC/HCFU; 52 weeks	1001	414	587	0.73 (0.57–0.95)
Akasu^14^	2006	UFT; 52 weeks	274	135	139	0.52 (0.33–0.81)
Sakamoto^40^	2004	5‐FU/MMC (Arm 7‐1‐C) UFT/MMC (Arm 7‐1‐R) HCFU/MMC (Arm 7–2) HCFU/MMC/FU IV (Arm 15); 52 weeks	5228	2458	2770	0.85 (0.77–0.93)
Kato^15^	2002	UFT; 104 weeks	289	144	145	‐
Zaniboni^16^	2000	5‐FU/LV; 24 weeks	869	446	423	0.66 (0.53–0.83)
O'Connell^17^	1997	5‐FU/LV; 24 weeks	309	151	158	‐
Ito^18^	1996	HCFU; 52 weeks	173	85	88	‐
IMPACT^19^	1995	5‐FU/LV; 24 weeks	1493	757	736	0.67 (0.56–0.80)
Moertel^20^	1995	5‐FU/levamisole; 48 weeks	929	315	614	0.59 (0.46–0.77)
The Colorectal Cancer Chemotherapy Group of Japan^21^	1995	5‐FU/MMC; 24 weeks	906	293	613	‐
Francini^22^	1994	5‐FU/LV; 48 weeks	234	118	116	0.65 (0.48–0.82)
Wolmark^41^	1988	Semustine/vincristine/5‐FU; 70 weeks	741	383	358	0.71 (0.39–0.97)
Stage IV						
Kanemitsu^38^	2021	mFOLFOX6; 24 weeks	300	149	151	0.67 (0.50–0.92)
Hasegawa^23^	2016	UFT/LV; 25 weeks	177	89	88	0.56 (0.38–0.83)
Portier^24^	2006	5‐FU/LV; 24 weeks	171	85	86	0.66 (0.46–0.96)
Kemeny^39^	2002	HAI‐FUDR and 5‐FU; 24 weeks	75	45	30	‐

### 3.2 RFS Dynamics in all randomized studies

**FIGURE 2:**
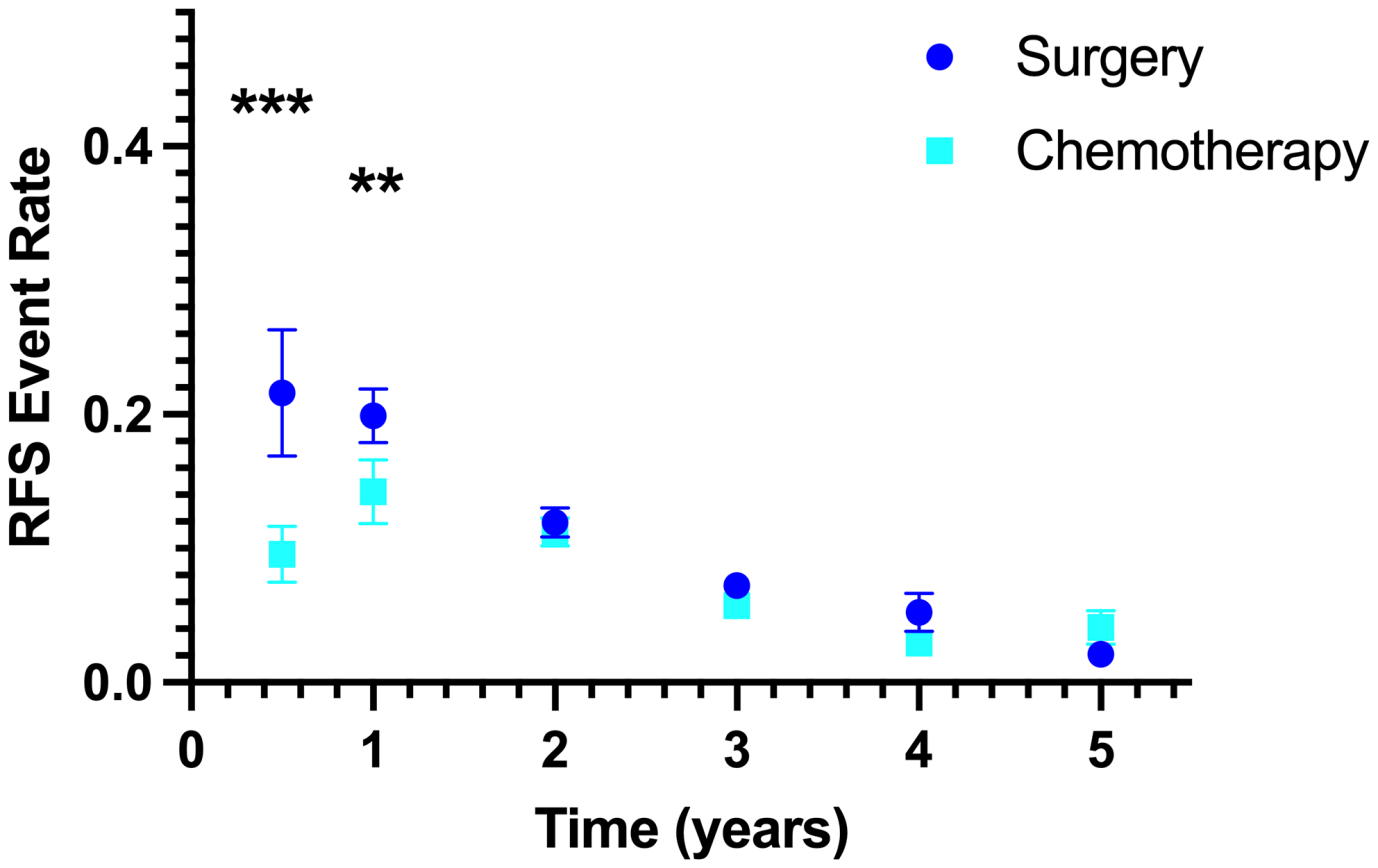
Event rates of estimated recurrence‐free survival (RFS) for randomized Phase III trials of adjuvant chemotherapy versus surgery alone for resected Stage II–IV colorectal cancer. Each point represents the mean slope with SEM. ***p < 0.001; **p < 0.01.

In the absence of adjuvant chemotherapy (*n* = 7819 patients), the greatest rate of recurrence was observed in the first year. There were similar mean RFS event rates for the interval 0–0.5 years (0.22 ± 0.21) and 0.5–1 years (0.20 ± 0.09), whereas the mean rate for RFS decreased every interval thereafter (Table 2). These observations align with a biological model of recurrence whereby there are two potential residual tumor cell populations, with most recurrences arising early from residual micrometastatic disease that remains in cell cycle, with less frequent recurrences arising years after resection from dormant tumor cells. One prediction of this model would be that adjuvant cytotoxic chemotherapy effects the active tumor‐cell population but not the dormant tumor‐cell population. In accordance with this prediction, in the overall cohort (*n* = 16,682 patients), we observed that the addition of adjuvant chemotherapy was associated with significant decreases in the RFS event rates for the intervals 0–0.5 years and 0.5–1 years after randomization (Figure 2). The mean event rate in the chemotherapy treatment arm during the first 0.5 years after randomization was approximately half that observed in the surgery control arm (0.09 ± 0.09 vs. 0.22 ± 0.21, *p* < 0.001), with a smaller difference in the mean RFS event rate for the interval 0.5–1 years (0.14 ± 0.11 vs. 0.20 ± 0.09, *p* = 0.001). There was no significant difference in RFS event rates at any of the later intervals evaluated.

**TABLE 2:** RFS event rates in the overall cohort (*n* = 16,682).

Interval (years)	Surgery [mean (SD)]	Surgery + adjuvant chemotherapy [mean (SD)]	*p*‐value
0–0.5	0.22 (0.21)	0.10 (0.10)	<0.001
0.5–1	0.20 (0.09)	0.14 (0.11)	0.001
1–2	0.12 (0.05)	0.11 (0.05)	0.553
2–3	0.07 (0.04)	0.06 (0.02)	0.071
3–4	0.05 (0.06)	0.03 (0.02)	0.118
4–5	0.02 (0.02)	0.04 (0.06)	0.147

### 3.3 RFS dynamics stratified by pathologic stage

Patients with resected oligometastatic CRC have an increased risk of recurrence. This heightened risk may be mediated by an increased pool of active tumor‐cell population, dormant tumor‐cell population, or both. We hypothesized that Stage IV patients have a larger active tumor‐cell population that may drive early recurrence, with no differences in the dormant tumor‐cell population that may drive late recurrences. To evaluate this in human trial data, the RFS event rates were individually examined for patients with localized (Stage II/III) versus metastatic (Stage IV) CRC who did not receive any adjuvant chemotherapy (*n* = 7451 and 368, respectively). Compared to localized CRC patients, those with resected metastatic CRC demonstrated faster RFS event rates for the early postoperative intervals of 0–0.5 years (0.56 ± 0.23 vs. 0.13 ± 0.08, *p* = 0.033) and 0.5–1 years (0.32 ± 0.05 vs. 0.17 ± 0.07, *p* = 0.003) (Table 3). There was no significant difference in RFS dynamics for these patients managed with surgery alone for the evaluated time intervals after 1 year from randomization.

**TABLE 3:** RFS event rates in the surgery only treatment arms, stratified by AJCC staging (*n* = 7819).

Interval (years)	Surgery Stage II/III [mean (SD)]	Surgery Stage IV [mean (SD)]	*p*‐value
0–0.5	0.13 (0.08)	0.56 (0.23)	0.033
0.5–1	0.17 (0.07)	0.32 (0.05)	0.003
1–2	0.11 (0.04)	0.15 (0.08)	0.485
2–3	0.08 (0.04)	0.04 (0.01)	0.052
3–4	0.05 (0.07)	0.06 (0.05)	0.875
4–5	0.02 (0.01)	0.03 (0.04)	0.807

**FIGURE 3:**
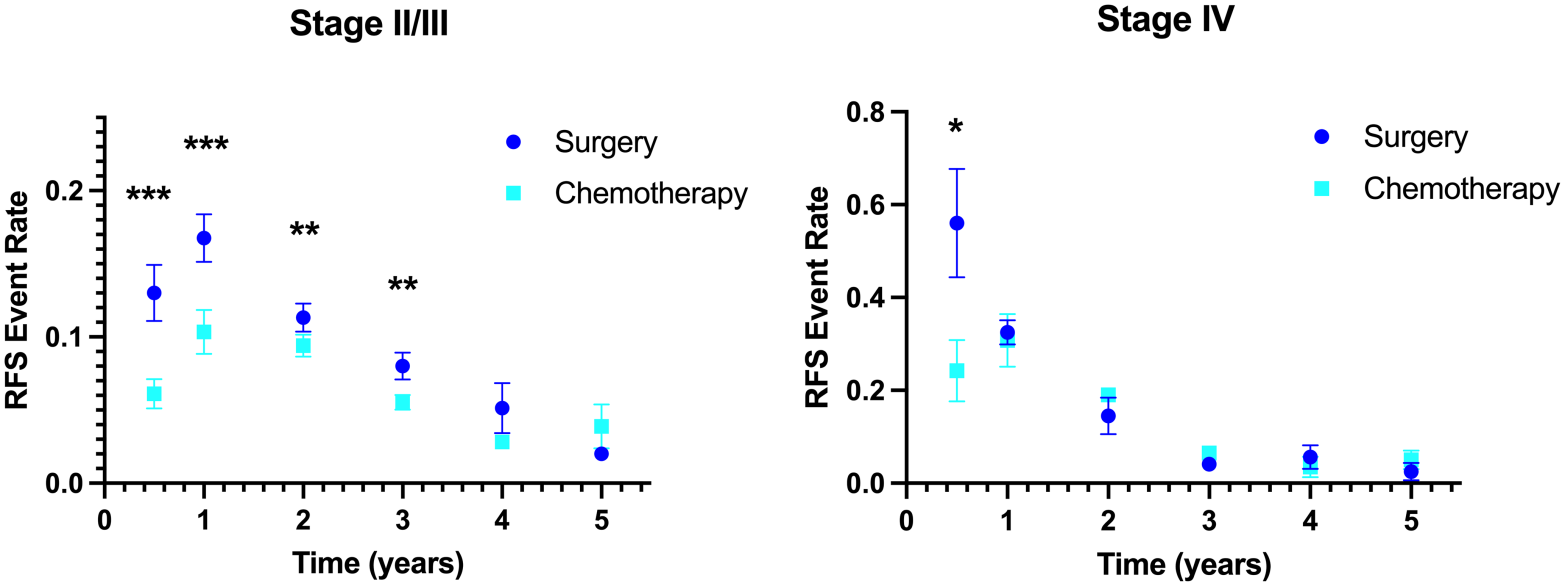
Event rates of estimated recurrence‐free survival (RFS) for randomized Phase III trials of adjuvant chemotherapy versus surgery alone for resected Stage II/III (left) and Stage IV (right) colorectal cancer. Each point represents the mean slope with SEM. ***p < 0.001; **p < 0.01; *p < 0.05.

The impact of adjuvant chemotherapy on RFS dynamics was then assessed separately for the subset of trials for localized and metastatic CRC. For trials involving Stage IV patients (*n* = 723), all of which included adjuvant regimens of 6 months duration, differences in the mean RFS event rates were only observed for the interval 0–0.5 years (0.24 ± 0.13 vs. 0.56 ± 0.23, *p* = 0.012) (Figure 3). For trials involving Stage II/III patients (*n* = 15,959), there were marked differences in the mean RFS event rates for the interval 0–0.5 year (0.06 ± 0.04 vs. 0.13 ± 0.08, *p* < 0.001) and the interval 0.5–1 year (0.10 ± 0.06 vs. 0.17 ± 0.07, *p* < 0.001), with smaller absolute differences for the intervals 1–2 years (0.09 ± 0.03 vs. 0.11 ± 0.04, *p* = 0.012) and 2–3 years (0.06 ± 0.2 vs. 0.08 ± 0.04, *p* = 0.010).

### 3.4 RFS dynamics stratified by length of chemotherapy treatment

**FIGURE 4:**
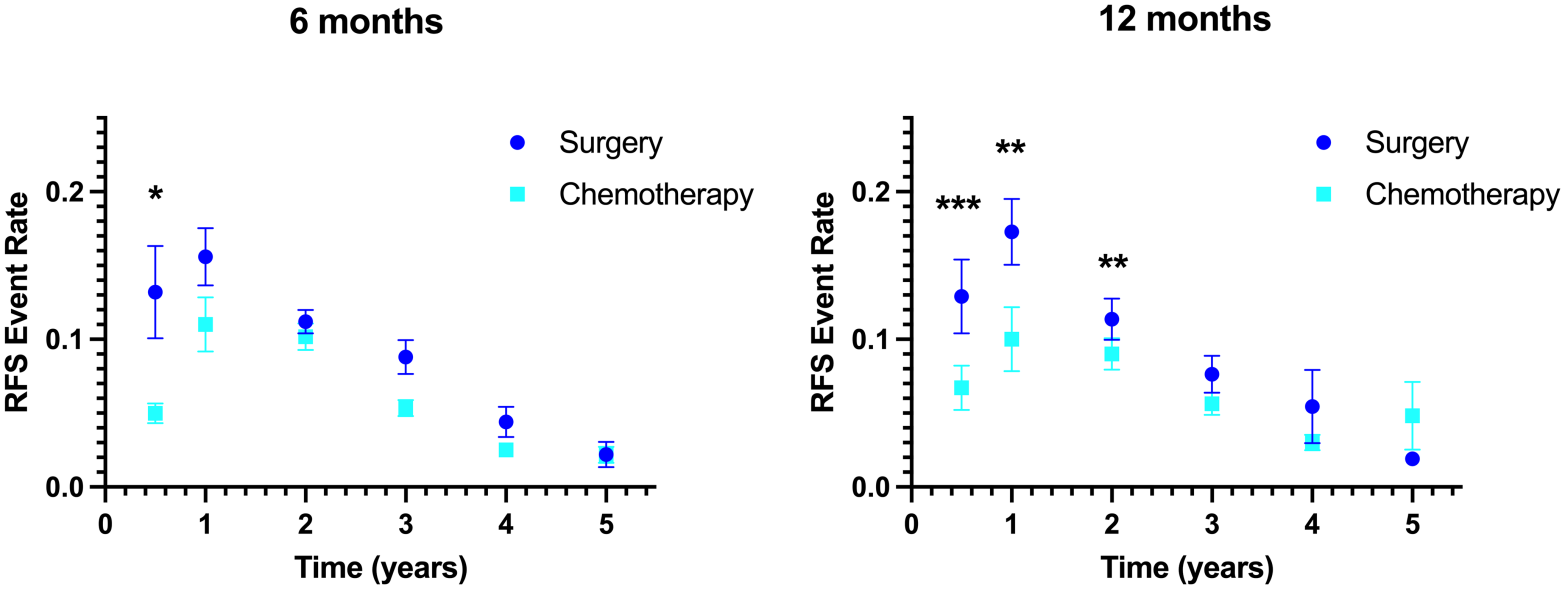
Event rates of estimated recurrence‐free survival (RFS) for randomized Phase III trials of adjuvant chemotherapy versus surgery alone for resected Stage II/III colorectal cancer, stratified by treatment duration. Each point represents the mean slope with SEM. ***p < 0.001; **p < 0.01; *p < 0.05.

In the randomized studies of Stage IV patients (*n* = 723), all patients randomized to the treatment arm received 6 months of adjuvant therapy, corresponding with the time interval where a significant difference in RFS event rates was observed. To further explore the relationship between RFS event rates and treatment duration, the randomized trials of Stage II/III patients were stratified by length of treatment. In this subset of trials where 6 months of adjuvant therapy was utilized in the treatment arm (*n* = 3552 patients), a significant difference in the RFS event rate was observed for the interval during treatment (0–0.5 years: 0.05 ± 0.02 vs. 0.13 ± 0.07, *p* = 0.031), with no significant differences at later time intervals (Figure 4). In the subset of trials where 12 months of adjuvant therapy was utilized in the treatment arm (*n* = 4453 patients), marked difference in RFS event rates were observed for the intervals during treatment (0–0.5 years: 0.07 ± 0.05 vs. 0.13 ± 0.08, *p* < 0.001; 0.5–1 years: 0.10 ± 0.07 vs. 0.17 ± 0.07, *p* = 0.001) as well as more modest differences in RFS events in the interval immediately following treatment completion (1–2 years: 0.09 ± 0.04 vs. 0.11 ± 0.05, *p* = 0.007), with no significant differences at later time intervals.

## 4 DISCUSSION

The benefit of adjuvant chemotherapy for selected populations with CRC is well‐established, with demonstrated efficacy to improve RFS.^8^ Three to six months of 5‐fluorouracil (5‐FU) with oxaliplatin has become the standard of care for adjuvant Stage IV, Stage III (node‐positive), as well as high‐risk Stage II disease with proficient mismatch repair proteins. However, the temporal dynamics of response to such therapy has not been systematically quantified. In this systematic review of published Phase III clinical trials with positive results and a surgery only arm, we explored real‐world recurrence dynamics to understand how active cytotoxic chemotherapy regimens work to improve RFS. We observed that, in the absence of adjuvant treatment, the greatest rate of recurrence occurred in the first year after curative‐intent surgery. Resected Stage IV patients demonstrated a faster recurrence rate during this early interval, with no differences as compared to Stage II/III patients at later time intervals. In this context, the addition of cytotoxic chemotherapy was associated with divergences in RFS event rates during—or shortly after—the administration of systemic chemotherapy, which was observed for both localized and metastatic patients. In contrast, late recurrence rates were not modified by the use of adjuvant chemotherapy. These data may provide in vivo understanding of residual tumor cell populations after curative‐intent resection and how chemotherapy can be best used to prevent recurrence.

First, analysis of RFS dynamics provides new understanding of the increased risk of recurrence for resected Stage IV CRC. We observed that the rate of recurrence for localized and metastatic CRC was remarkably similar 1 year after surgical resection. The marked differences in overall recurrence rates (estimated 30% vs. 80% for localized and metastatic CRC, respectively^1^, ^2^) were driven by rapid recurrence rates for Stage IV CRC patients in the first 12 months after randomization. One possible explanation is that stage IV patients harbor a larger population of proliferating residual micrometastatic cells. In the GALAXY study, part of the CIRCULATE‐Japan project, postoperative circulating tumor DNA (ctDNA) was used as a measurable biomarker of residual micrometastatic cells that are proliferating and shedding DNA fragments. Here, the rate of ctDNA positivity increased with increasing pathologic stage, from approximately 6% in node‐negative Stage I–II patients, 23% in node‐positive Stage III patients, and 30% in Stage IV patients.^25^ Similarly, in studies of circulating tumor cells (CTCs), another presumed biomarker of proliferating micrometastatic cells, rates of CTC detection increased with disease stage.^29^ Many of the trial patients included in this systematic review received care prior to the development of ctDNA technologies, and thus their postoperative MRD status is not known; nevertheless, these trial data along with modern MRD studies provide complementary understanding on recurrence dynamics. In particular, these data could be explained by a model of cancer recurrence whereby *early* recurrences are driven by a micrometastatic cell population in a proliferative state that shed ctDNA.

This model of recurrence, where there is an early, proliferating micrometastatic population, predicts the observed benefit of adjuvant cytotoxic chemotherapy. In support, this analysis of recurrence dynamics found a temporal relationship between duration of therapy and disease treatment. In our analysis of RCTs where 6 months of adjuvant therapy were utilized, improvements in the RFS event rates were limited to the first 6 months of surveillance. In RCTs where 12 months of adjuvant therapy were used, the most significant differences in RFS event rates were again observed during the treatment intervals. If the presence of ctDNA is a biomarker of this proliferating and chemotherapy‐sensitive micrometastatic population, then one may expect that it can help tailor selection for adjuvant therapy, and serial monitoring may help identify those who may benefit from extended therapy. In support, the recently published GALAXY study demonstrated the adjuvant chemotherapy was associated with improved RFS for patients with detectable ctDNA but was not associated with RFS improvements for the ctDNA‐negative cohort.^25^ The ongoing CIRCULATE‐US study (NRG‐GI008) is exploring these findings in a prospective, randomized trial in which detection of postoperative ctDNA guides the use and intensity of adjuvant chemotherapy.^26^

In contrast, late recurrence rates were not significantly different among treatment and control patients in this systematic review of randomized studies. This may be explained by a process of recurrence where dormant (and treatment‐refractory, in the case of the included adjuvant therapies in this analysis) cells stochastically resume proliferation.^27^, ^28^ Several quiescent cancer cell lines—including colorectal—have indeed proven chemotherapy‐resistant both in vitro and in vivo compared to their actively proliferating counterparts.^9^, ^30^, ^31^, ^32^ In this case, residual disease remains quiescent and undetectable via routine surveillance until environmental or intracellular cues trigger proliferation, resulting in clinical recurrence or metastasis.^3^ This latent reservoir of dormant cells has been termed drug‐tolerant persisters (DTPs).^6^, ^9^ These data together suggest that the evaluated adjuvant chemotherapy regimens cannot be expected to improve late recurrences, and suggest alternative strategies that may involve targeting dormant DTPs.^33^, ^34^

These findings have important implications for the duration of systemic chemotherapy for resected CRC. We observed RFS event rates were greatest in the first 6 months after surgery, providing rationale for the current duration for adjuvant treatment of 3–6 months and highlight the importance of early initiation of adjuvant therapy. The rapidly diminishing RFS event rates at time intervals further from surgery suggest a shrinking population of proliferating and chemotherapy‐sensitive micrometastases. If efficacy of adjuvant chemotherapy is related to the recurrence rate during time of chemotherapy administration, and baseline recurrence rates drops off exponentially, then extending chemotherapy will have diminishing returns, and the absolute effect of extending therapy may be modest.

While there were significant differences in RFS event rates observed for the duration of systemic therapy, including beyond 1 year for those trials of 12 months of adjuvant therapy, treatment duration must balance diminishing efficacy with the effects of systemic toxicity and its impact on quality of life.^35^, ^36^ In the pooled analysis of six randomized trials comparing 3–6 months of adjuvant chemotherapy (the IDEA consortium), which demonstrated that a shorter duration of adjuvant therapy is as effective for most patients, it is likely that brief chemotherapy is effective in managing this rapidly diminishing pool of proliferating cells after surgery. One exception from the IDEA trials is that 6 months of adjuvant fluorouracil, leucovorin, and oxaliplatin (FOLFOX) may be preferred in high‐risk patients, as defined by T4 and/or N2 disease.^35^ Here, advanced pathologic features may be a marker of a greater risk of proliferating micrometastases that benefit from therapeutic eradication. In support, in an exploratory analysis of 2010 patients from one of the IDEA trials (IDEA‐France), ctDNA‐positive patients had significantly worse RFS when treated with the 3‐month regimen.^37^ In the future, assessments of ctDNA and other methods of minimal residual disease detection will hopefully allow for more targeted application of cytotoxic chemotherapy.

Several limitations warrant emphasis. Primarily, heterogeneity among the included studies is a weakness inherent to all systematic reviews. There was considerable variation among the RCTs included in our analysis which were conducted across multiple countries and individually enrolled 75–5233 patients.^38^, ^39^, ^40^, ^41^ There were small variations in the protocolized assessments of disease recurrence. Similarly, the included RCTs also spanned several decades, with resultant advances in the imaging quality of assessment for recurrence. There is also risk of heterogeneous disease biology in the study populations among the included studies, with differences in clinical, pathological or molecular characteristics that may impact recurrence rates.^42^ Still, such a reliable relationship between duration of therapy and observed RFS dynamics, despite these sources of heterogeneity, supports the real‐world clinical applicability of this biologic observation. Another limitation is the exclusion in this systematic review of trials utilizing a perioperative chemotherapy strategy, such as the EORTC 40983.^43^ Hence, these results cannot be extrapolated to other sequencing strategies of systemic chemotherapy for CRC. Third, the included studies of extended adjuvant chemotherapy relied on UFT‐based regimens, which may have limited the effectiveness of these regimens. However, the use of more robust chemotherapeutic regimens for extended adjuvant therapies is likely limited by treatment‐related toxicities and would not be supported by these recurrence dynamics rates. Fourth, these results should be interpreted in the context of cytotoxic chemotherapy and may or may not apply for targeted therapy and/or immunotherapies, with their potentially different effects on proliferating vs. dormant micrometastatic cells. Relatedly, future analyses will be needed to explore RFS dynamics among trials where two chemotherapy regimens were utilized, without a surgery‐only control arm. These current data help answer an important question about the natural history of recurrence for resected CRC, where a surgery‐only control was necessary. Lastly, this analysis represents a systematic review with attention to RFS event dynamics and it bears emphasizing that it is not a meta‐analysis and thus does not exhaustively include all studies (i.e., both positive and negative) and does not estimate the effect size for adjuvant chemotherapy (as a pooled hazard ratio).

## 5 CONCLUSION

We present an evaluation of temporal recurrence dynamics in pooled analysis of positive Phase III RCTs of adjuvant chemotherapy for Stage II–IV CRC. Adjuvant cytotoxic chemotherapy improved early but not remote recurrence dynamics over surgery‐only controls. This was observed for both nonmetastatic as well as oligometastatic CRC populations, and the effect was primarily limited to duration of therapy. These results support ongoing evaluation of ctDNA technologies to guide adjuvant treatment decisions and improved understanding of dormancy for rationale therapeutic strategies to prevent late recurrence.

## AUTHOR CONTRIBUTIONS

**Emma Vail:** Conceptualization (equal); data curation (lead); formal analysis (equal); investigation (equal); methodology (equal); project administration (equal); writing – original draft (lead); writing – review and editing (equal). **Ankur P. Choubey:** Data curation (supporting); project administration (supporting); writing – original draft (supporting); writing – review and editing (equal). **H. Richard Alexander:** Writing – review and editing (equal). **David A. August:** Writing – review and editing (equal). **Abril Berry:** Data curation (equal); resources (equal); software (equal); writing – review and editing (equal). **Patrick M. Boland:** Writing – review and editing (equal). **Mariam F. Eskander:** Writing – review and editing (equal). **Miral S. Grandhi:** Writing – review and editing (equal). **Brittany Haliani:** Data curation (equal); investigation (equal); resources (equal); software (equal); writing – review and editing (equal). **Haejin In:** Writing – review and editing (equal). **Timothy J. Kennedy:** Writing – review and editing (equal). **Russell C. Langan:** Writing – review and editing (equal). **Jason C. Maggi:** Writing – review and editing (equal). **Henry A. Pitt:** Writing – review and editing (equal). **Shridar Ganesan:** Conceptualization (equal); formal analysis (equal); funding acquisition (lead); methodology (equal); project administration (equal); resources (equal); validation (equal); writing – review and editing (equal). **Brett L. Ecker:** Conceptualization (lead); data curation (equal); formal analysis (equal); investigation (equal); methodology (equal); project administration (equal); resources (equal); software (lead); supervision (lead); writing – original draft (equal); writing – review and editing (equal).

## FUNDING INFORMATION

SG is supported by grants from the NCI (RO1 CA243547, PO1CA250957, RO1CA233662, P30CA072720), DoD (W81XWH‐20‐BCRP‐BTA12‐2), Gertrude Fogarty Trust, AHEPA and NJCCR.

## CONFLICT OF INTEREST STATEMENT

SG has consulted for Merck, Roche, MD Serano, EQRX, Foghorn Therapeutics, KayoThera, Foundation Medicine, Ceptur Therapeutics and has research funding from Gandeeva Therapeutics and M2GEN. His spouse is an employee of Merck and has equity in Merck. RL has consulted for Eon.

## ETHICS STATEMENT

This study is IRB‐exempt due to the publicly available nature of the data.

## Supporting information


Data S1.

## Data Availability

The data supporting study findings are available from the corresponding author upon reasonable request.
