# Supplementary material for: Recurrence‐free survival dynamics following adjuvant chemotherapy for resected colorectal cancer: A systematic review of randomized controlled trials
Source: Cancer Med. 2024 Jan 8;13(1):e6884. doi: 10.1002/cam4.6884 (PMC10807601; doi:10.1002/cam4.6884)
Supplement: Supplementary file 1 — Data S1. [file CAM4-13-e6884-s001.docx]

**PubMed Search Strategy**

MeSH Terms:

#1 randomized trial

#2 adjuvant

#3 chemotherapy

#4 colon cancer

#5 rectal cancer

#6 adenocarcinoma

#7 colorectal

#8 phase III randomized controlled trial

1. Search: **colorectal cancer AND phase III randomized controlled trial** Filters: **Randomized Controlled Trial, English**

(("colorectal neoplasms"[MeSH Terms] OR ("colorectal"[All Fields] AND "neoplasms"[All Fields]) OR "colorectal neoplasms"[All Fields] OR ("colorectal"[All Fields] AND "cancer"[All Fields]) OR "colorectal cancer"[All Fields]) AND (("phase"[All Fields] OR "phase s"[All Fields] OR "phases"[All Fields]) AND "III"[All Fields] AND ("randomized controlled trial"[Publication Type] OR "randomized controlled trials as topic"[MeSH Terms] OR "randomized controlled trial"[All Fields] OR "randomised controlled trial"[All Fields]))) AND ((randomizedcontrolledtrial[Filter]) AND (english[Filter]))

**Translations**

**colorectal cancer:** "colorectal neoplasms"[MeSH Terms] OR ("colorectal"[All Fields] AND "neoplasms"[All Fields]) OR "colorectal neoplasms"[All Fields] OR ("colorectal"[All Fields] AND "cancer"[All Fields]) OR "colorectal cancer"[All Fields]

**phase:** "phase"[All Fields] OR "phase's"[All Fields] OR "phases"[All Fields]

**randomized controlled trial:** "randomized controlled trial"[Publication Type] OR "randomized controlled trials as topic"[MeSH Terms] OR "randomized controlled trial"[All Fields] OR "randomised controlled trial"[All Fields]

2. Search: **Colon cancer OR rectal cancer OR adenocarcinoma OR colorectal AND phase iii randomized controlled trial** Filters: **Randomized Controlled Trial, English**

(("colonic neoplasms"[MeSH Terms] OR ("colonic"[All Fields] AND "neoplasms"[All Fields]) OR "colonic neoplasms"[All Fields] OR ("colon"[All Fields] AND "cancer"[All Fields]) OR "colon cancer"[All Fields] OR ("rectal neoplasms"[MeSH Terms] OR ("rectal"[All Fields] AND "neoplasms"[All Fields]) OR "rectal neoplasms"[All Fields] OR ("rectal"[All Fields] AND "cancer"[All Fields]) OR "rectal cancer"[All Fields]) OR ("adenocarcinoma"[MeSH Terms] OR "adenocarcinoma"[All Fields] OR "adenocarcinomas"[All Fields] OR "adenocarcinoma s"[All Fields]) OR "colorectal"[All Fields]) AND (("phase"[All Fields] OR "phase s"[All Fields] OR "phases"[All Fields]) AND "iii"[All Fields] AND ("randomized controlled trial"[Publication Type] OR "randomized controlled trials as topic"[MeSH Terms] OR "randomized controlled trial"[All Fields] OR "randomised controlled trial"[All Fields]))) AND ((randomizedcontrolledtrial[Filter]) AND (english[Filter]))

**Translations**

**Colon cancer:** "colonic neoplasms"[MeSH Terms] OR ("colonic"[All Fields] AND "neoplasms"[All Fields]) OR "colonic neoplasms"[All Fields] OR ("colon"[All Fields] AND "cancer"[All Fields]) OR "colon cancer"[All Fields]

**rectal cancer:** "rectal neoplasms"[MeSH Terms] OR ("rectal"[All Fields] AND "neoplasms"[All Fields]) OR "rectal neoplasms"[All Fields] OR ("rectal"[All Fields] AND "cancer"[All Fields]) OR "rectal cancer"[All Fields]

**adenocarcinoma:** "adenocarcinoma"[MeSH Terms] OR "adenocarcinoma"[All Fields] OR "adenocarcinomas"[All Fields] OR "adenocarcinoma's"[All Fields]

**phase:** "phase"[All Fields] OR "phase's"[All Fields] OR "phases"[All Fields]

**randomized controlled trial:** "randomized controlled trial"[Publication Type] OR "randomized controlled trials as topic"[MeSH Terms] OR "randomized controlled trial"[All Fields] OR "randomised controlled trial"[All Fields]

3. Search: **colon cancer OR rectal cancer OR adenocarcinoma OR colorectal AND phase iii randomized controlled trial**

("colonic neoplasms"[MeSH Terms] OR ("colonic"[All Fields] AND "neoplasms"[All Fields]) OR "colonic neoplasms"[All Fields] OR ("colon"[All Fields] AND "cancer"[All Fields]) OR "colon cancer"[All Fields] OR ("rectal neoplasms"[MeSH Terms] OR ("rectal"[All Fields] AND "neoplasms"[All Fields]) OR "rectal neoplasms"[All Fields] OR ("rectal"[All Fields] AND "cancer"[All Fields]) OR "rectal cancer"[All Fields]) OR ("adenocarcinoma"[MeSH Terms] OR "adenocarcinoma"[All Fields] OR "adenocarcinomas"[All Fields] OR "adenocarcinoma s"[All Fields]) OR "colorectal"[All Fields]) AND (("phase"[All Fields] OR "phase s"[All Fields] OR "phases"[All Fields]) AND "iii"[All Fields] AND ("randomized controlled trial"[Publication Type] OR "randomized controlled trials as topic"[MeSH Terms] OR "randomized controlled trial"[All Fields] OR "randomised controlled trial"[All Fields]))

**Translations**

**colon cancer:** "colonic neoplasms"[MeSH Terms] OR ("colonic"[All Fields] AND "neoplasms"[All Fields]) OR "colonic neoplasms"[All Fields] OR ("colon"[All Fields] AND "cancer"[All Fields]) OR "colon cancer"[All Fields]

**rectal cancer:** "rectal neoplasms"[MeSH Terms] OR ("rectal"[All Fields] AND "neoplasms"[All Fields]) OR "rectal neoplasms"[All Fields] OR ("rectal"[All Fields] AND "cancer"[All Fields]) OR "rectal cancer"[All Fields]

**adenocarcinoma:** "adenocarcinoma"[MeSH Terms] OR "adenocarcinoma"[All Fields] OR "adenocarcinomas"[All Fields] OR "adenocarcinoma's"[All Fields]

**phase:** "phase"[All Fields] OR "phase's"[All Fields] OR "phases"[All Fields]

**randomized controlled trial:** "randomized controlled trial"[Publication Type] OR "randomized controlled trials as topic"[MeSH Terms] OR "randomized controlled trial"[All Fields] OR "randomised controlled trial"[All Fields]
